# Epidemiological survey of scoliosis screening in schools among 51,025 adolescents in Gannan Tibetan autonomous prefecture, Gansu Province, China

**DOI:** 10.3389/fpubh.2026.1693960

**Published:** 2026-01-20

**Authors:** Dong Hou, Shaobo Yang, Jing Zhang, Yan Zhang, Fangjun Yang, Chen Zhang, Jin Huang, Changquan Dai, Juan Wang, Xiaoyun Yuan, Xiaojuan Cui, Kai Li, Han Leng, Jun Zhao, Jiantao Wen

**Affiliations:** 1 School of Integrated Chinese and Western Medicine, Gansu University of Chinese Medicine, Lanzhou, China; 2 The First Clinical Medical College, Nanjing University of Chinese Medicine, Nanjing, China; 3 Gansu Provincial Hospital of Traditional Chinese Medicine, Lanzhou, China; 4 Longnan First People's Hospital, Longnan, China

**Keywords:** adolescents, epidemiology, prevalence, risk factors, scoliosis

## Abstract

**Background:**

We completed a school suspected scoliosis screening (SSS) of multi-ethnic adolescents in Gannan Tibetan Autonomous Prefecture, Gansu Province, China, to examine the prevalence of suspected scoliosis in this area and associated risk factors.

**Methods:**

From October 2024 to April 2025, we conducted scoliosis screening (SSS) in eight districts of the Gannan Tibetan Autonomous Prefecture: Zhaoni, Lintan, Luqu, Diebu, Zhouqu, Maqu, Xiahe, and Hezuo. Visual examination, the Adams forward bending test (FBT), and trunk rotation angle measurement were used to recognize suspected scoliosis. In this study, all screening-positive results are based on trunk rotation angle (ATR) ≥ 5, defined as “Suspected Scoliosis.” Demographic data were gathered, and the prevalence of suspected scoliosis was determined. The Least Absolute Shrinkage and Selection Operator (LASSO) regression technique was employed to identify characteristics most correlated with suspected scoliosis. The selected variables were further analyzed using multivariate logistic regression to ascertain the correlation between suspected scoliosis and the related factors.

**Results:**

A total of 67 schools and 51,025 adolescents (24,821 males and 26,204 females) attended screening. The total prevalence of suspected scoliosis among adolescents in the Gannan Tibetan Autonomous Prefecture was 2.37% (1,211 cases), with a prevalence of 2.76% in females, higher than the 1.97% seen in males (*p* < 0.01). LASSO regression analysis revealed a substantial association between age, height, weight, BMI, and latitude and suspected scoliosis. Following the exclusion of extraneous variables through LASSO regression, multivariate logistic regression analysis indicated that age (OR = 1.054, 95% CI: 1.027–1.082), gender (OR = 1.395, 95% CI: 1.241–1.567), BMI (OR = 0.950, 95% CI: 0.927–0.974), and latitude (OR = 0.611, 95% CI: 0.531–0.703) were independently correlated with suspected scoliosis. According to ATR, severity was categorized into three grades: Grade I suspected scoliosis in 832 cases (68.71%), Grade II suspected scoliosis in 296 instances (24.44%), and Grade III suspected scoliosis in 83 cases (6.83%). The thoracic segment, thoracolumbar segment, and lumbar segment comprised 25.85, 32.04, and 42.11%, respectively. No significant variations were seen in the distribution of suspected scoliosis severity across various segments.

**Conclusion:**

The prevalence of suspected scoliosis among multi-ethnic students in Gannan Tibetan Autonomous Prefecture was 2.37%. Age, gender, BMI, and latitude were identified as factors influencing the prevalence.

## 1 Introduction

Adolescent scoliosis is a three-dimensional structural deformity of the spine characterized by aberrant vertebral alignment in the frontal, lateral, and axial planes. Diagnosis can be established by assessing the Cobb angle in the coronal plane via X-ray, with a measurement of ≥10° (1, 2). Scoliosis can be categorized into neuromuscular scoliosis, idiopathic scoliosis, congenital scoliosis, and scoliosis resulting from other factors, depending on the etiology (3). Idiopathic scoliosis (IS) is the predominant subtype, comprising roughly 80% of prevalence, and is especially common in teenagers aged 10 to 18 years. International epidemiological research indicate that the global prevalence of scoliosis varies between 0.47 and 5.2% (4). A cross-sectional survey of 99,695 children in mainland China indicated an overall scoliosis prevalence of 5.14% (5). Individuals with mild or moderate scoliosis generally exhibit no symptoms and may remain undetected. If overlooked and mistreated, it may result in severe scoliosis and deformity, frequently necessitating surgical intervention in advanced stages. Consequently, school screenings are vital for facilitating early intervention and averting disease advancement.

School-based scoliosis screening (SSS) is an important tool for the early detection and prediction of spinal curvature in adolescents and the identification of high-risk populations for this condition (6). SSS encompasses various assessment methods, with the FBT and ATR measurement being the most commonly used methods for identifying scoliosis patients, as they are widely accepted for their high sensitivity (71.1%) and specificity (97.1%) (7). Additionally, these methods are easy to implement in large-scale screenings due to their simplicity and lack of radiation risks. Furthermore, early intervention with treatment measures (including observation and follow-up, rehabilitation exercises, orthotic braces, and surgical intervention) can effectively control disease progression and prevent long-term complications resulting from worsening spinal deformities, such as respiratory dysfunction, impaired motor function, chronic low back pain, postural abnormalities, and reduced quality of life (8–11).

In China, disparities in medical conditions, geographical location, climatic factors, and lifestyle habits between the eastern and western areas lead to differing prevalence of scoliosis. Research investigation indicates that the prevalence of scoliosis is marginally greater in the northern region (1.3%) compared to the southern region (1.2%) (12). The prevalence of scoliosis in Chinese teenagers rose from 0.9% from 2000–2015 to 1.6% from 2016 to 2024 (13). The prevalence of scoliosis varies with altitude compared to low-altitude areas. Ethnic minorities frequently inhabit isolated regions characterized by comparatively low population densities. Research reveals that the prevalence of scoliosis in Tibetan children residing on the Qinghai-Tibet Plateau is 3.69%, whereas the rates among Han and Tibetan populations in Tianzu Tibetan Autonomous County, Gansu Province, are 10.8 and 7.1%, respectively (14, 15). Presently, disparities exist in the prevalence of scoliosis across ethnic minorities; however, data concerning these communities are scarce. Consequently, Chinese health officials deem it essential to advocate for scoliosis screening, specifically focusing on teenagers aged 10 to 18 as the core demographic for screening. Epidemiological surveys can enhance comprehension of scoliosis prevalence and its distinct characteristics. Nonetheless, comprehensive epidemiological assessments are now absent in Gannan Tibetan Autonomous Prefecture, China. Moreover, there is insufficient evidence regarding the prevalence of scoliosis in the Gannan region and other elevated areas. This study aims to examine the prevalence of scoliosis and associated risk factors among multi-ethnic school-aged kids in the Gannan region.

## 2 Materials and methods

### 2.1 Subjects and study design

Gannan Tibetan Autonomous Prefecture is one of China’s ten Tibetan autonomous prefectures, where the Tibetan population constitutes 56.99% of the entire local population (16). Gannan is situated on the northeastern periphery of the Qinghai-Tibet Plateau, with an average altitude of 2,960 meters. The severe environmental conditions in this region, characterized by low air pressure, diminished oxygen levels, frigid temperatures, and intense radiation, have resulted in distinctive cultural traditions, lifestyles, dietary habits, and a comparatively low socioeconomic status (17, 18). The Gannan region possesses a modest population, notable geographical changes, distinctive topography, a high density of ethnic minorities, and relatively underdeveloped economic and medical infrastructure, accompanied by limited scientific research activities. Consequently, we designated Gannan Tibetan Autonomous Prefecture as the research area for this study. The Ethics Committee of Gansu Provincial Hospital of Traditional Chinese Medicine has authorized the study (2023-048-01). All procedures involving human subjects in this study adhere to the ethical standards established by the 1975 Declaration of Helsinki and its revisions, together with the ethical criteria set forth by the relevant institutional and/or national research committees. As the majority of participants are minors, informed permission forms have been signed by all parents or guardians, who have been apprised of the study.

The research was carried out from October 2024 until April 2025. We performed a scoliosis screening in 67 educational institutions, comprising elementary, junior high, high, ethnic, and vocational schools, across eight regions of Gannan Tibetan Autonomous Prefecture: Zhaoni, Lintan, Luqu, Diebu, Zhouqu, Maqu, Xiahe, and Hezuo. The screening regions are situated between 100°45′ and 104°45′ east longitude, 33°06′ and 35°34′ north latitude, with heights varying from 1,172 to 4,920 meters. A total of 51,025 students (24,821 males and 26,204 females) participated in this survey, encompassing 17 ethnic groupings.

The screening procedure in this study adhered rigorously to the national standardized protocols specified in the “Clinical Practice Guidelines and Pathway Guidelines for Adolescent Scoliosis Screening in China (2021).” A standardized multidisciplinary team was established, comprising senior orthopedic spine surgeons, clinical nurses, rehabilitation therapists, and graduate research assistants. All screening personnel had undergone formal training and possessed at least 1 year of experience in school-based scoliosis screening, ensuring specialized expertise in the field.

All subjects completed a visual examination and the Adams FBT. Initially, throughout the visual assessment, the examinee was directed to maintain a natural erect stance with feet aligned closely together. The examiner meticulously assessed and documented the horizontal alignment of the shoulders, symmetry of the scapulae, symmetry of the waist, and any pelvic inclination. Secondly, the examinee is directed to bend forward at the waist, lower their head to gaze at their toes, extend their elbows and knees, relax their shoulders, position their hands in front of their knees, and maintain their feet together (19). The Adams FBT is conducted to assess thoracic rotation, scapular asymmetry, pelvic curvature, and spinous processes, while reducing the impact of non-structural scoliosis (20). To minimize inter-observer variability and measurement bias, a rigorous tiered screening protocol was implemented. Specifically, all participants identified as positive during the initial visual inspection and Adams FBT were subject to a final confirmatory examination by a lead senior spine surgeon prior to the measurement of the ATR. This expert-led verification was designed to minimize diagnostic errors and ensure the integrity of the collected data.

**Figure 1:**
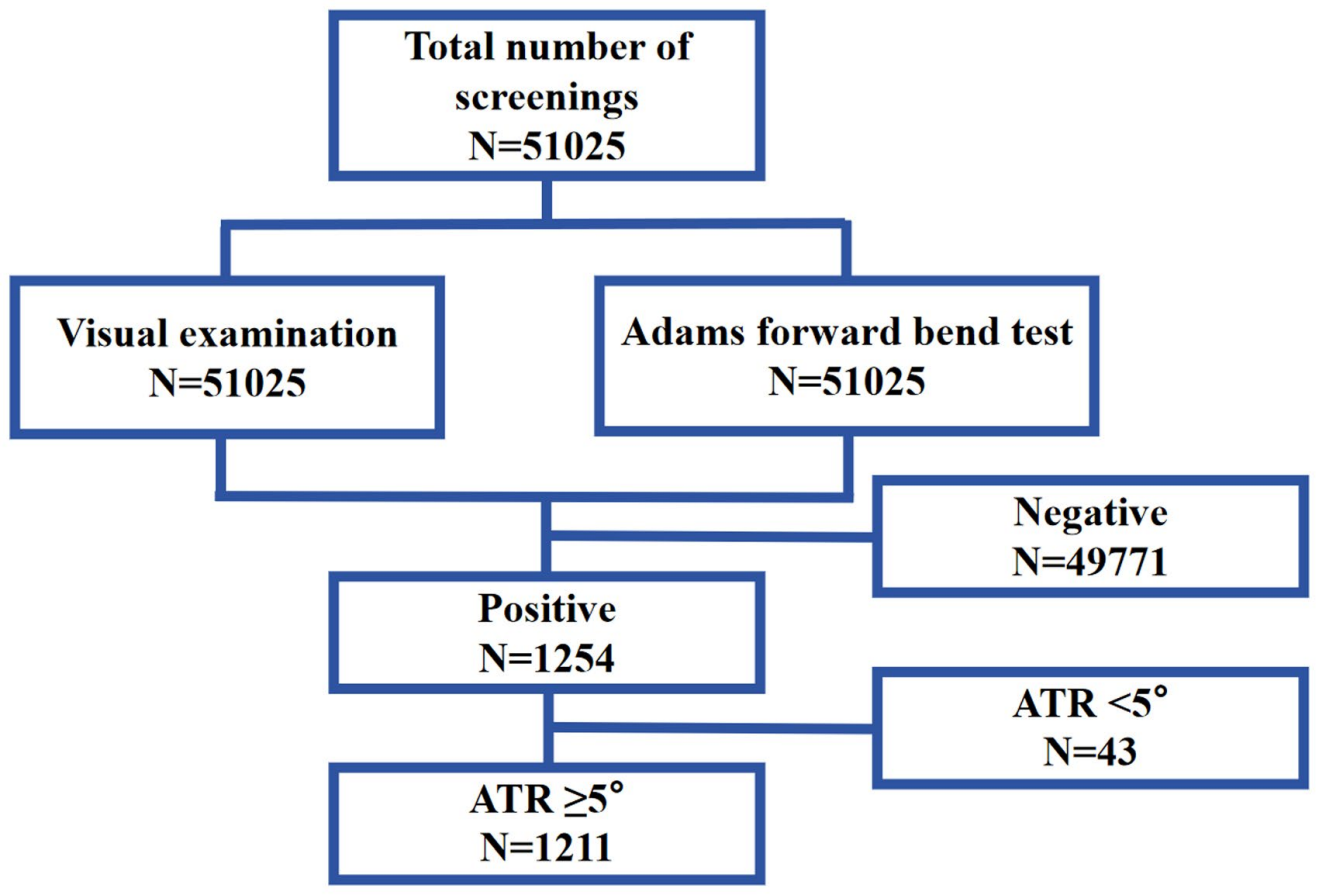
Participants flow diagram.

Subsequently, all examinees exhibiting positive results from ocular inspection or the Adams FBT receive assessment of ATR. Utilize a portable electronic scoliosis scanner (model: SpineScan SH-105) to scan the spine from the cervical region downward, documenting the highest ATR value as it traverses the upper thoracic, thoracolumbar, and lumbar vertebrae. Simultaneously, examinees directed to participate in ATR testing were required to execute spinal motions, following which ATR testing was reiterated to document the outcomes subsequent to the movements. If both ATR readings were ≥5°, this was deemed prevalence of suspected scoliosis (Figure 1). Suspected scoliosis patients were instructed to have their instructors and parents accompany them to a local or specialized hospital for standing full-spine anteroposterior and lateral X-ray examinations.

### 2.2 Data collection

Following the completion of the ATR measurements, we gathered screening information forms and noted the number of students who took the test. Names, gender, age, ethnicity, height (m), weight (kg), and ATR (°) were all given on these forms. We also noted the school’s location’s average elevation, latitude, longitude, and annual average temperature. Using the formula weight (kg) divided by height (m) squared, we determined their body mass index (BMI) based on their height and weight. All of the information gathered was promptly arranged in spreadsheets for further examination.

### 2.3 Diagnostic criteria

Following ocular inspection and affirmative results from the Adams FBT, we advanced to the assessment of the ATR. Previous studies indicate that ATR values of 5° and 7° correlate to Cobb angles of 11° and 20°, respectively, as assessed from full-length spinal X-rays (2, 21). Consequently, we formulated a diagnostic criterion for suspected scoliosis as two successive ATR measures of ≥5°. Patients with suspected scoliosis were categorized into three categories according to ATR magnitude: Grade I (5° ≤ ATR < 7°), Grade II (7° ≤ ATR < 10°), and Grade III (ATR ≥ 10°) (22).

### 2.4 Statistical analysis

All analyses were conducted utilizing SPSS 27.0 (IBM Corporation, USA) and R software (version 4.4.3). Continuous variables are presented as mean ± standard deviation (SD), and independent samples *t*-tests were employed to compare groups. Categorical variables are represented as frequency and percentage [*n* (%)], and chi-square tests were employed for intergroup comparisons. Pearson correlation coefficients were employed to evaluate the association between various parameters potentially influencing the prevalence of suspected scoliosis, determining the strength and direction of linear correlations among these variables.

Whereafter, we employed the LASSO method for initial variable selection. The LASSO regression model identified height, weight, and BMI as key factors influencing the prevalence of suspected scoliosis. We then performed classical logistic regression analysis using the selected variables.

Since BMI is mathematically derived as weight divided by height squared (BMI = weight/height^^2^), the inherent collinearity among height, weight, and BMI can lead to incorrect estimation of model parameters and biased outcomes during regression analysis. Although LASSO regression helps mitigate multicollinearity during variable selection, interpreting the included, highly correlated variables remains statistically challenging. Therefore, we chose to exclude height and weight, which were collinear with BMI, to ensure the accuracy and stability of the final logistic regression model. This approach leveraged the advantages of logistic regression to provide more accurate, interpretable, and valid insights into the relationship between BMI and suspected scoliosis.

Based on the results of the LASSO regression, we excluded the collinearity effects of height and weight on BMI and developed a multifactorial logistic regression model to examine the factors influencing the prevalence of suspected scoliosis, using odds ratios (OR) and 95% confidence intervals (CI). Statistical significance was defined as *p* < 0.05 (bilaterally). This approach ensures that the final model includes only the most relevant predictors, providing a more robust and clinically interpretable analysis of the risk factors for suspected scoliosis.

## 3 Results

### 3.1 SSS screening for geographic and demographic characteristics

**Figure 2:**
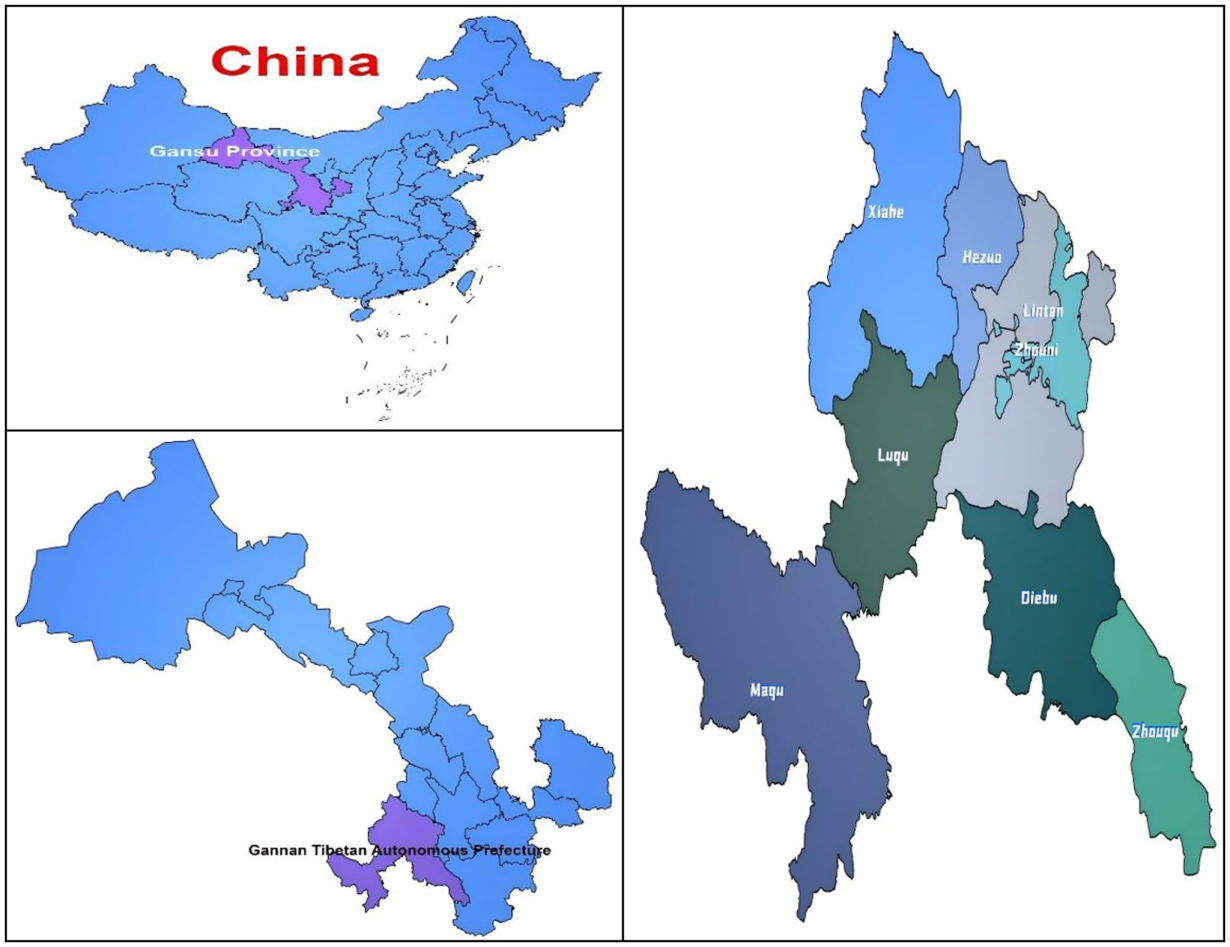
This study examines the geographical locations of the eight counties involved.

**Figure 3:**
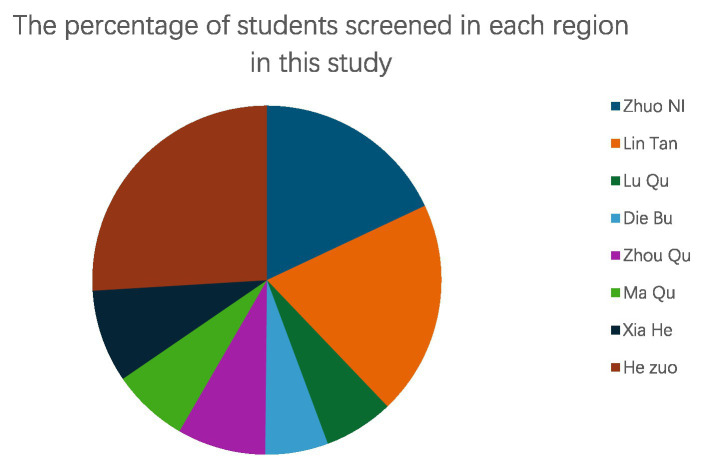
Proportion of students evaluated in each region within this study.

This research examined 67 schools and 51,025 pupils aged 10 to 18, comprising 24,821 boys (48.64%) and 26,204 girls (51.36%). They originated from several areas within the Gannan Tibetan Autonomous Prefecture, such as Zhaoni County, Lintan County, Luqu County, Diebu County, Zhouqu County, Maqu County, Xiahe County, and Hezuo City, among others. The geographical attributes and the quantity of pupils assessed in each region differed (Figures 2, 3).

**Figure 4:**
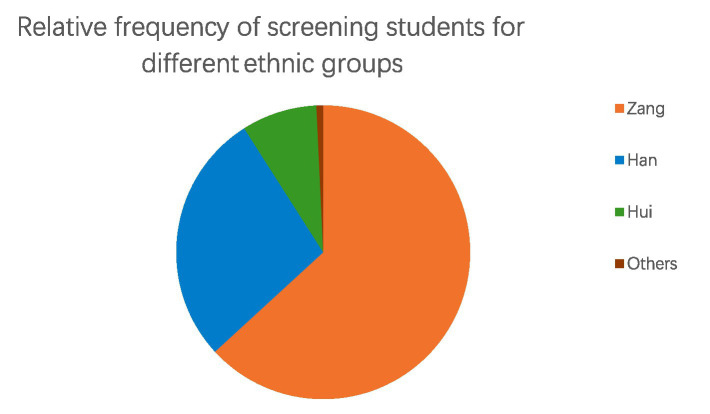
Proportional frequency of screening students among ethnic groups.

Among the 16 ethnic groups in the total screening population, Tibetans, Han, and Hui account for 99% of the total population (Figure 4).

### 3.2 Summary of suspected scoliosis screening

**Figure 5:**
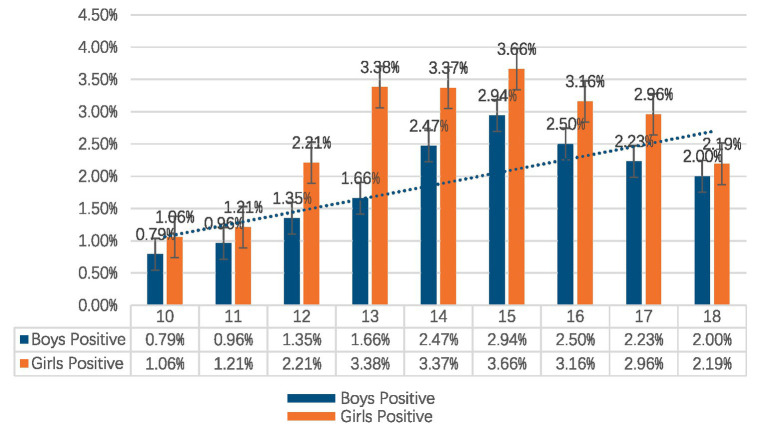
Relative frequency of suspected scoliosis categorized by age group and stratified by gender.

The aggregate count of probable adolescent suspected scoliosis cases was 1,211, yielding a prevalence of 2.37%. The prevalence rate for girls was 2.76%, above that of boys at 1.97%. There is a significant difference in the prevalence between males and females aged 12–14. The prevalence of suspected scoliosis escalated with age, especially between 10 and 15 years, in both males and females. The prevalence of suspected scoliosis in girls began to rise progressively from ages 11 to 12 (2.21%) and peaked at ages 15 (3.66%). The prevalence for males progressively rose from 1.66% at years 12–13 to a peak of 2.94% at ages 15 (Table 1 and Figure 5).

**Table 1:** Positive distribution results of suspected scoliosis screening in students stratified by gender.

Age	Boys			Girls			Chi-square	*p* value
	*N*	Suspected scoliosis screening positive	Positive rate %	*N*	Suspected scoliosis screening positive	Positive rate %		
10	2033	16	0.79%	2083	22	1.06%	0.815	0.367
11	2,598	25	0.96%	2,477	30	1.21%	0.733	0.392
12	2,812	38	1.35%	2,854	63	2.21%	5.929	0.015
13	3,372	56	1.66%	3,398	115	3.38%	20.422	<0.001
14	3,683	91	2.47%	3,684	124	3.37%	5.208	0.022
15	3,811	112	2.94%	3,905	143	3.66%	3.156	0.076
16	3,198	80	2.50%	3,512	111	3.16%	2.629	0.105
17	2067	46	2.23%	2,605	77	2.96%	2.399	0.121
18	1,247	25	2.00%	1,686	37	2.19%	0.125	0.724
Total	24,821	489	1.97%	26,204	722	2.76%	33.918	<0.001

The analysis of the characteristics of individuals with positive and negative scoliosis screening results, including gender, height, weight, and BMI, shows a significant association between suspected scoliosis and gender, weight, and BMI. Adolescent girls and those with lower weight and BMI are more likely to be screened as suspected scoliosis cases. Although there is a difference in height between the two groups, the difference is not significant (Table 2).

**Table 2:** Demographic characteristics of screening positive and negative individuals.

Characteristic	Suspected scoliosis screening positive (*n* = 1,211)	Suspected scoliosis screening negative (*n* = 49,814)	*p* value
Gender			
Boys	489 (40.38)	24,332 (48.85)	<0.001
Girls	722 (59.62)	25,482 (51.15)	
Height (mean ± SD, m)	1.61 ± 10.33	1.62 ± 10.78	0.067
Weight (mean ± SD, kg)	46.33 ± 8.33	48.51 ± 10.36	<0.001
BMI (mean ± SD, kg/m^2^)	17.71 ± 2.25	19.48 ± 3.41	<0.001

**Figure 6:**
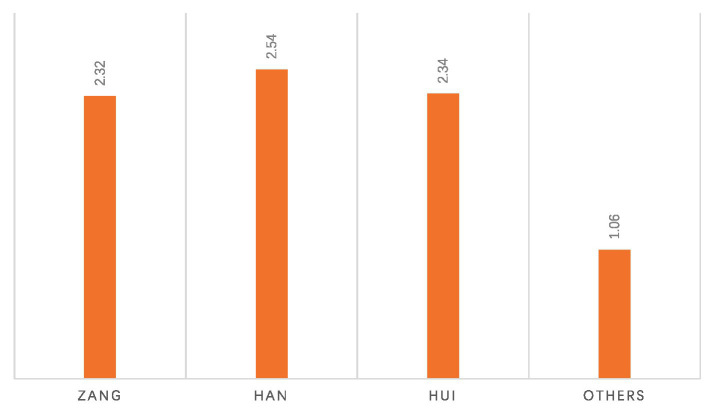
Prevalence of suspected scoliosis by ethnicity: positive screening rate.

Analysis of the suspected scoliosis results across different ethnicities and regions in Gannan showed that ethnic factors have a minimal impact on suspected scoliosis, with no significant differences observed. Among them, the suspected scoliosis prevalence rate is 2.32% in the Tibetan population, 2.54% in the Han population, 2.34% in the Hui population, and 1.06% in other ethnic groups (Figure 6). However, there were significant differences in the positive screening rates for suspected scoliosis across regions, with Maqu and Diebu counties having higher positive rates of 3.93 and 3.63%, respectively (Table 3).

**Table 3:** Prevalence of suspected scoliosis among students stratified by ethnicity and region.

Variable	Category	*N*	Suspected scoliosis screening positive	Positive rate %	*p* value
Ethnicity	Zang	32,231	747	2.32	0.167
	Han	14,187	361	2.54	
	Hui	4,229	99	2.34	
	Others	378	4	1.06	
County	Zhuo Ni	9,186	107	1.17	<0.001
	Lin Tan	10,135	276	2.72	
	Lu Qu	3,297	89	2.70	
	Die Bu	2,975	108	3.63	
	Zhou Qu	4,182	127	3.04	
	Ma Qu	3,613	142	3.93	
	Xia He	4,388	87	1.98	
	He Zuo	13,249	275	2.08	

### 3.3 ATR grading of suspected scoliosis and distribution of suspected scoliosis sites

ATR classifies severity into three distinct grades: Grade I (5° ≤ ATR < 7°), Grade II (7° ≤ ATR < 10°), and Grade III (ATR ≥ 10°) (22). A total of 1,211 patients with suspected scoliosis were included in the study. Among these, 832 cases (68.71%) were classified as Grade I suspected scoliosis, 296 cases (24.44%) as Grade II suspected scoliosis, and 83 cases (6.83%) as Grade III suspected scoliosis. The thoracic segment, thoracolumbar segment, and lumbar segment represented 25.85, 32.04, and 42.11%, respectively. No notable differences were observed in the distribution of suspected scoliosis severity across various segments (Table 4).

**Table 4:** ATR grading of suspected scoliosis and distribution of suspected scoliosis sites.

ATR grade	Thoracic segment	Thoracolumbar segment	Lumbar segment	Total number	*χ* ^2^	*p*
Grade I	198 (16.36%)	272 (22.46%)	362 (29.89%)	832 (68.71%)	7.075	0.132
Grade II	86 (7.10%)	92 (7.60%)	118 (9.74%)	296 (24.44%)		
Grade III	29 (2.40%)	24 (1.98%)	30 (2.47%)	83 (6.85%)		

### 3.4 Correlation analysis and LASSO regression

**Figure 7:**
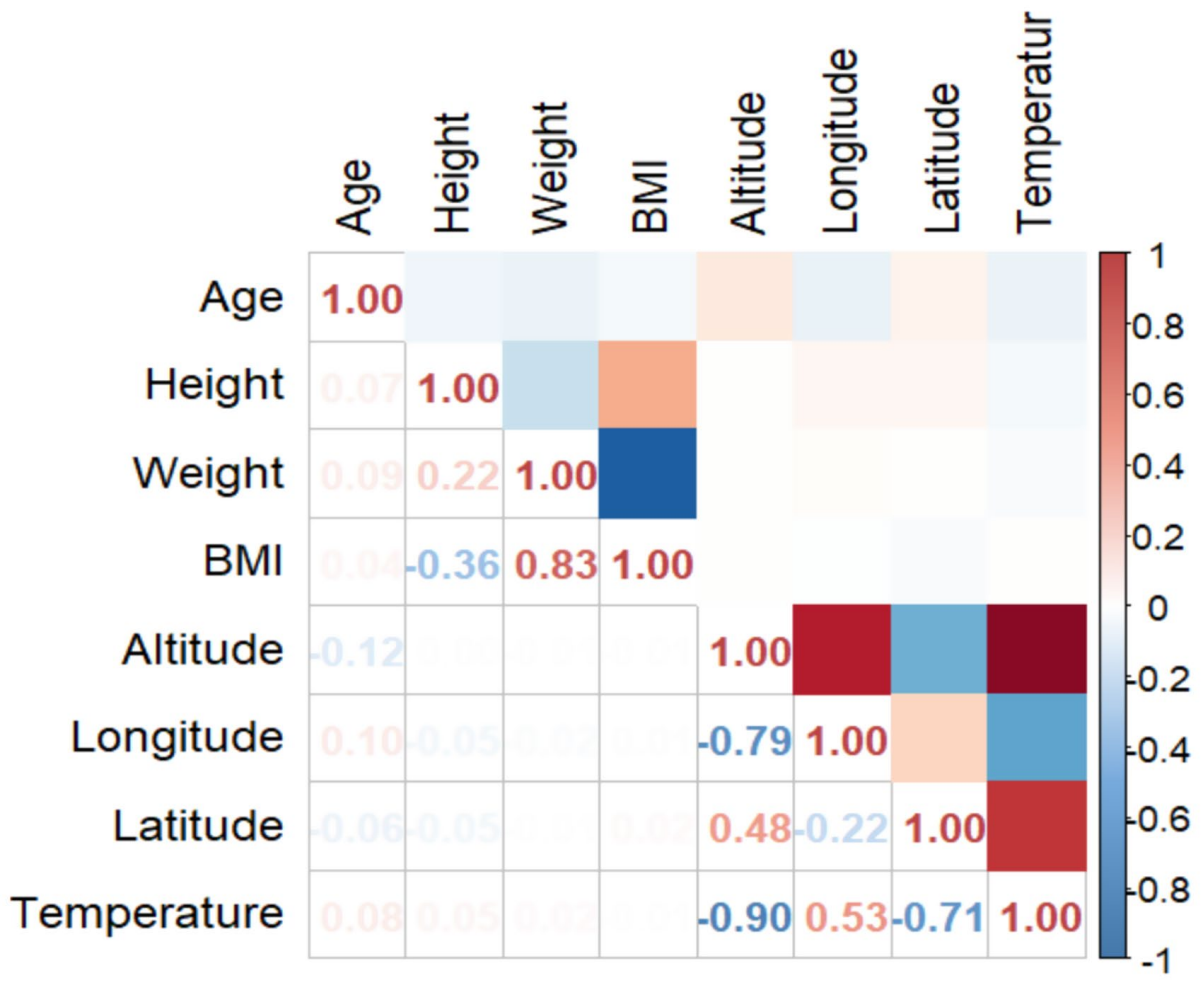
Correlation matrix diagram of pertinent variables.

Performed a correlation analysis to evaluate the relationships between different factors that could impact the prevalence of suspected scoliosis. The variables that showed correlation were age, height, weight, BMI, altitude, longitude, latitude, and temperature. The correlation matrix indicated multiple noteworthy relationships among the study variables (Figure 7). The correlation between BMI and weight was found to be strong and positive (*R*^2^ = 0.83). In contrast, altitude exhibited a strong negative correlation with both longitude and temperature (*R*^2^ = −0.79, *R*^2^ = −0.90). Additionally, latitude showed a moderate negative correlation with temperature (*R*^2^ = −0.71).

The dependent variable in this study was suspected scoliosis, while the independent factors comprised age, height, weight, BMI, altitude, longitude, latitude, and temperature. Initially, 10-fold cross-validation was employed to compute the mean squared error (MSE) of the model across various *λ* values, helping identify the optimal λ value. This process allowed for the selection of the most relevant predictors while mitigating overfitting.

Subsequently, through the examination of the LASSO regression coefficient trajectories, we observed that as the penalty parameter (log lambda) increased, the coefficients for altitude, longitude, and temperature diminished to zero, leading to their exclusion from the final model. By applying L1 regularization, LASSO reduced the influence of redundant variables, allowing for a more robust and interpretable model.

**Figure 8:**
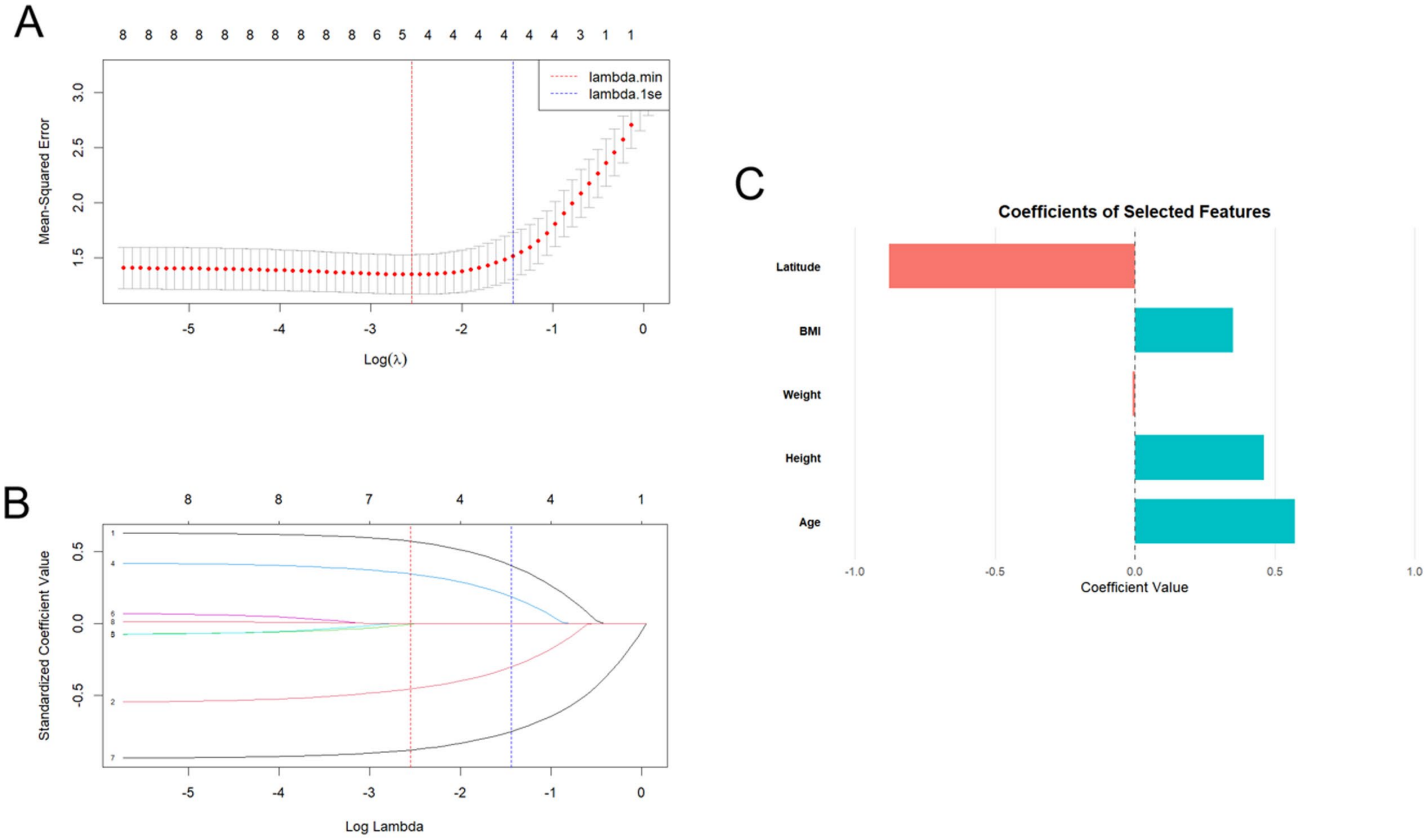
**(A)** Cross-validation curve for LASSO regression: the solid line denotes the mean cross-validation error, whereas the region between the two dashed lines signifies the positive and negative standard deviation range of log(*λ*). The left dashed line represents the log(λ) value at which model error is minimized. **(B)** Plot of the LASSO coefficient path: variables are enumerated as follows: 1–Age, 2–Height, 3–Weight, 4–Body mass index (BMI), 5–Altitude, 6–Longitude, 7–Latitude, 8–Temperature. As λ escalates, standardized coefficients are constrained towards zero. When a coefficient is regularized to zero, it signifies that the corresponding variable has been excluded from the model. The longer a variable’s coefficient is constrained to zero, the more significant its impact on the model. At the ideal log(λ) = −2.55391, five variables were identified. **(C)** Coefficients for the five chosen variables.

As a result, age, height, weight, BMI, and latitude showed significant associations with suspected scoliosis, suggesting that these factors play a critical role in forecasting the prevalence of suspected scoliosis (Figure 8).

### 3.5 Multivariate logistic regression of the factors associated with suspected scoliosis in the unadjusted full model and the adjusted final model

Due to the collinearity between height, weight, and BMI, height and weight were excluded in the subsequent multi-factor regression analysis, while BMI was retained as a comprehensive measure of body composition to reduce the analytical errors caused by collinearity.

Multiple regression analysis revealed that age, gender, BMI, altitude, longitude, latitude, and temperature significantly influenced the prevalence of suspected scoliosis in the unadjusted full model (Table 5). The odds ratios (OR) and 95% confidence intervals (CI) are presented below: Age (OR = 1.085, 95% CI: 1.058–1.113), gender (OR = 1.395, 95% CI: 1.241–1.568), BMI (OR = 0.958, 95% CI: 0.935–0.982), altitude (OR = 1.004, 95% CI: 1.004–1.005), longitude (OR = 3.347, 95% CI: 2.675–4.187), latitude (OR = 1.857, 95% CI: 1.400–2.470), temperature (OR = 1.841, 95% CI: 1.688–2.008), Han (OR = 1.599, 95% CI: 1.383–1.849).

**Table 5:** Multivariate logistic regression analysis of suspected factors related to suspected scoliosis in the unadjusted complete model and adjusted final model.

Variable		Unadjusted full model				Adjusted final model			
		*p* value	OR	95%CI		*p* value	OR	95%CI	
				Lower	Upper			Lower	Upper
	Age	<0.001	1.085	1.058	1.113	<0.001	1.054	1.027	1.082
Gender	Boys (Reference)								
	Girls	<0.001	1.395	1.241	1.568	<0.001	1.395	1.241	1.567
	BMI	<0.001	0.958	0.935	0.982	<0.001	0.950	0.927	0.974
	Altitude	<0.001	1.004	1.004	1.005				
	Longitude	<0.001	3.347	2.675	4.187				
	Latitude	<0.001	1.859	1.400	2.470	<0.001	0.611	0.531	0.703
	Temperature	<0.001	1.841	1.688	2.008				
Ethnicity	Zang (Reference)								
	Han	<0.001	1.599	1.383	1.849	0.105	1.111	0.978	1.262
	Hui	0.010	1.340	1.072	1.675	0.129	0.953	0.959	1.467
	Others	0.379	0.641	0.238	1.726	0.196	0.521	0.194	1.399

OR, odds ratio; 95% CI, confidence interval for OR.

Following the LASSO regression variable selection, we excluded altitude, longitude, and temperature from the model to ensure the reliability and validity of the results, reducing the potential for model overfitting and enhancing scientific interpretation. Consequently, we performed an additional multivariate regression analysis using the adjusted final model. In the final adjusted model, we identified multiple factors influencing the prevalence of suspected scoliosis, including age, gender, BMI, latitude and ethnicity. The odds ratios (OR) and 95% confidence intervals (CI) for the final model are as follows: Age (OR = 1.054, 95% CI: 1.027–1.082), gender (OR = 1.395, 95% CI: 1.241–1.567), BMI (OR = 0.950, 95% CI: 0.927–0.974), and latitude (OR = 0.611, 95% CI: 0.531–0.703).

## 4 Discussion

This study provides valuable insights into the prevalence of suspected adolescent scoliosis in the multi-ethnic Gannan Tibetan Autonomous Prefecture, addressing a significant gap in the literature concerning this underrepresented region in northwestern China. The identified prevalence of 2.37% and key associated factors (e.g., age, gender, BMI, latitude) not only characterize the local epidemiology but also invite deeper exploration into the potential biomechanical, endocrine, and environmental mechanisms that may underlie these associations in such a specific high-altitude, multi-ethnic setting.

The prevalence of suspected scoliosis in Gannan Tibetan Autonomous Prefecture exhibits notable variation when compared to other regions. It is higher than rates reported in several countries, including the United States (0.2%) (10), Japan (0.87%) (23), India (0.61%) (24), and Singapore (1.37 and 2.22%) (25), but lower than another report from Japan (7.6%) (26). Within China, the prevalence in Gannan is higher than that in Hangzhou, Zhejiang (2.23%) (27) and Jinghong, Yunnan (1.74%) (22), but lower than that in the Qinghai-Tibet Plateau (3.69%) (14) and Shanghai (6.9%) (28). This substantial variation underscores that scoliosis epidemiology is not uniform but is modulated by a complex interplay of factors. Beyond demographic composition, divergent screening methodologies (e.g., ATR thresholds, examiner training) likely contribute to reported differences.

More fundamentally, geographical gradients, such as the higher prevalence observed on the Qinghai-Tibet Plateau compared to lower-altitude Yunnan sites—may point to the influence of altitude-related factors like vitamin D synthesis or growth patterns. Similarly, the high prevalence in metropolitan Shanghai could reflect lifestyle factors including intense academic pressure and reduced physical activity, which may impact para-spinal muscle development and spinal loading. These observations argue for regionally tailored screening strategies that consider local environmental and socio-behavioral contexts.

Our findings align with the established paradigm of adolescent scoliosis, showing a peak in suspected cases around age 15 and a consistent female predominance (1:1.40) (29). The gender difference is likely multifactorial. A key mechanistic hypothesis centers on endocrine physiology: Estrogen not only accelerates the pace of the pubertal growth spurt but may also influence the asynchronous development between the rapidly elongating spine and the surrounding muscular and ligamentous support structures, potentially compromising spinal stability during this vulnerable period (30, 31). Furthermore, the tendency for boys to engage in more physical exercise than girls is a factor that may promote more symmetrical development of the para-spinal musculature and offer a protective biomechanical effect.

Despite the multi-ethnic composition of the region, our analysis did not identify ethnicity as an independent risk factor for suspected scoliosis. This contrasts with some previous studies in other Tibetan areas of China which reported ethnic differences (15, 22). The discrepancy highlights the complexity of using broad ethnic categories as epidemiological variables. “Ethnicity” often serves as a proxy for a constellation of unmeasured or poorly measured factors, including genetic predisposition, socioeconomic status, nutritional practices, cultural norms around posture and physical activity, and health-seeking behaviors. The lack of association in our study suggests that within the specific context of Gannan, these underlying risk determinants may be distributed similarly across ethnic groups, or that their effects cancel out. This underscores the need for future research to move beyond ethnic labels and directly measure the specific biological, environmental, and behavioral factors that may confer risk or protection.

Our data support the importance of growth and body composition, revealing that adolescents with suspected scoliosis had significantly lower average weight and BMI compared to their normal peers. The study by Qi et al. indicated a higher detection rate of scoliosis among primary and middle school students who had lower body weight and greater height (32). The analysis of results by Brazolino et al. (33) has shown that adolescents with scoliosis had a lower body mass than normal. Spinal abnormalities are more readily identifiable in students with lower body weight, whereas in obese individuals, the accumulation of back fat may interfere with the accuracy of the Adams FBT and the measurement of the ATR.

As a comprehensive measure of body composition, BMI captures the net effect of both height and weight on the risk of scoliosis. Therefore, BMI emerged as the most meaningful predictor in our final model. Extensive research have also confirmed that a low BMI has a greater impact on both the prevalence and severity of scoliosis (34–37). A population-based prospective study demonstrated a negative correlation between BMI at ages 10 and 15 and scoliosis, finding that each one standard deviation increase in BMI was associated with a 20% reduction in the risk of scoliosis (38). BMI can reflect the nutritional status. Worthington et al. (39) indicated that malnutrition might play a crucial part in the etiology of scoliosis. A deficiency in key nutrients and low mechanical loading associated with reduced body mass can impair bone mineral density and vertebral development, compromising spinal stability. The study by Normand et al. found that patients with scoliosis exhibited insufficient or deficient serum levels of Vitamin D, a condition closely related to their nutritional status (40).

Other research indicated the association between lower BMI and scoliosis might be brought on by the interactions between multiple hormones, including leptin and adiponectin, which may influence bone metabolism and growth (41, 42). Leptin has been shown to be crucial in bone formation and remodeling, and low levels may inhibit normal vertebral growth and increase susceptibility to scoliosis progression (43). The study by Man et al. (44) clearly demonstrated the aberrant response of scoliosis osteoblasts to proliferation, differentiation, and mineralization when exposed to different concentrations of leptin. The latest research indicates that the reduced expression of leptin receptors in scoliosis may lead to low sensitivity to leptin in this condition (45). When systemic estrogen levels are low, the reduction in osteoblast differentiation may be accompanied by an increase in osteoclast formation, activation, and lifespan, consequently accelerating bone resorption (46). Currently, there are two main viewpoints regarding the involvement of estrogen in the pathogenesis of scoliosis. Abnormal estrogen levels lead to delayed menarche in females, subsequently retarding skeletal development and maturation, which increases the susceptibility to spinal deformity. Abnormal estrogen levels directly influence bone metabolism and remodeling, resulting in abnormal skeletal growth and development, thus increasing the probability of scoliosis (47). We thus hypothesize that low BMI serves as a reliable clinical indicator of a compromised metabolic and hormonal status that contributes to the pathogenesis of adolescent idiopathic scoliosis.

Based on these findings, a lower BMI may be interpreted as an indicator of nutritional insufficiency, which can negatively impact bone mineral density and muscle mass, consequently contributing to an increased prevalence of scoliosis and the aggravation of spinal deformity. This insight might facilitate the identification of high-risk populations with suspected scoliosis, thereby allowing for the implementation of preventive measures to delay or mitigate the onset and progression in subjects with low BMI.

Surveys of geographic data from various regions have found that latitude is a significant risk factor affecting the prevalence of suspected scoliosis. Although our study was limited to latitudes between 33°06′ and 35°34′, a study by Zhou et al. (1) in Dali, Yunnan, indicated that latitude significantly influences the prevalence of potential scoliosis. Higher latitude generally correlates with reduced ultraviolet B exposure, leading to lower endogenous synthesis of vitamin D. Vitamin D deficiency can impair calcium homeostasis and bone mineralization, potentially affecting vertebral strength and growth symmetry (48, 49). Concurrently, the photoperiod variation with latitude influences pineal gland secretion of melatonin. Altered melatonin levels have been hypothetically linked to disrupted control of skeletal growth and muscle tone, which could predispose to spinal deformity. An integrative hypothesis posits that latitude, through its effects on both the vitamin D and melatonin pathways, may modulate the endocrine milieu during the critical period of adolescent growth, thereby influencing scoliosis risk.

Early school screening is an important method for the timely identification and intervention of scoliosis. An ATR ≥ 5° is considered a basis for auxiliary scoliosis screening. Currently, Cobb angle measurement is the gold standard for assessing the severity of scoliosis (50). However, repeated exposure to radiation poses certain risks to the body, making ATR a non-invasive, radiation-free, direct, and practical measurement method. Numerous studies have confirmed that ATR ≥ 5° has acceptable sensitivity and specificity in predicting scoliosis (51, 52). Given the limitations and risks of routine radiographic screening in children, especially concerns about the increased risk of breast cancer in young females due to cumulative radiation exposure (53), most screening programs in China use ATR ≥ 5° as the referral criterion. Du et al. (54) conducted scoliosis screening on 57,393 primary and secondary school students in Qingdao, finding a strong positive correlation between ATR and the radiological Cobb angle.

The scoliometer serves as a screening instrument that exhibits acceptable levels of sensitivity and specificity for the detection of suspected scoliosis cases. Because ATR is ethically preferable for large-scale screening due to the avoidance of radiation, it is widely utilized, but it is not diagnostic. Therefore, the prevalence reported here is a “suspected screening positivity rate,” which may overestimate the true prevalence of radiographically confirmed scoliosis. Consequently, the identified risk factors are associated with the likelihood of screening positive. Their relationship with the severity or progression of radiographically confirmed curves may differ. This methodological framework is shared by many large-scale school screening programs in China and allows for efficient identification of at-risk adolescents for secondary clinical evaluation. Nonetheless, it necessitates caution when directly comparing our prevalence figures with studies using different screening thresholds or diagnostic gold standards, and when extrapolating etiological mechanisms from screening-based associations.

### 4.1 Limitations and future directions

Several limitations should be acknowledged. First, the cross-sectional design precludes causal inference regarding the identified risk factors. Second, as noted above, the use of ATR-based screening without routine radiographic confirmation means our estimates reflect screening suspected positivity rather than confirmed disease prevalence, and etiological associations may be attenuated or altered. Third, while we adjusted for several key variables, residual confounding from unmeasured factors (e.g., detailed physical activity levels, dietary habits, genetic markers, specific socioeconomic indicators) is possible. Fourth, the study is geographically focused on Gannan Prefecture, which limits generalizability to other populations with different genetic, environmental, and cultural backgrounds, although it provides crucial baseline data for this understudied region.

Future research should prioritize longitudinal cohorts with radiographic follow-up of ATR-positive individuals to establish true prevalence and progression rates, and to validate risk factors against diagnostic outcomes. Incorporating advanced measures of body composition, hormonal assays, genetic screening, and detailed environmental exposure assessment will be essential to move beyond associations and toward understanding the mechanistic pathways leading to scoliosis in vulnerable adolescents.

## 5 Conclusion

In summary, this large-scale school screening initiative provides crucial baseline data for Gannan Tibetan Autonomous Prefecture, revealing a suspected scoliosis prevalence of 2.37% among multi-ethnic adolescents. Our study shows a complex association between anthropometric indicators and screening positivity. Specifically, the BMI of the screening-positive group was lower, and the multivariate model further confirmed that a higher BMI was independently associated with a lower risk, highlighting the importance of body composition dynamics over simple body-size indicators. In addition, the association with latitude supports an integrative hypothesis that environmental factors may exert influence through Vitamin D and melatonin pathways. These results elucidate potential high-risk characteristics in this understudied multi-ethnic population, which can guide the efficient deployment of secondary clinical evaluations and the formulation of targeted public health strategies.

## Data Availability

The raw data supporting the conclusions of this article will be made available by the authors, without undue reservation.

## References

[1] Zhou J Wang Y Xie J Zhao Z Shi Z Li T . Scoliosis school screening of 139,922 multi-ethnic children in Dali, southwestern China: a large epidemiological study. iScience. (2023) 26:108305. doi: 10.1016/j.isci.2023.108305, 38025787 PMC10679892

[2] Negrini S Donzelli S Aulisa AG Czaprowski D Schreiber S de Mauroy JC . 2016 SOSORT guidelines: orthopaedic and rehabilitation treatment of idiopathic scoliosis during growth. Scoliosis Spinal Disord. (2018) 13:3. doi: 10.1186/s13013-017-0145-8, 29435499 PMC5795289

[3] El-Hawary R Chukwunyerenwa C. Update on evaluation and treatment of scoliosis. Pediatr Clin N Am. (2014) 61:1223–41. doi: 10.1016/j.pcl.2014.08.007, 25439021

[4] Konieczny MR Senyurt H Krauspe R. Epidemiology of adolescent idiopathic scoliosis. J Child Orthop. (2013) 7:3–9. doi: 10.1007/s11832-012-0457-4, 24432052 PMC3566258

[5] Hengwei F Zifang H Qifei W Weiqing T Nali D Ping Y . Prevalence of idiopathic scoliosis in Chinese schoolchildren: a large, population-based study. Spine (Phila Pa 1976). (2016) 41:259–64. doi: 10.1097/brs.0000000000001197, 26866739

[6] Scaturro D de Sire A Terrana P Costantino C Lauricella L Sannasardo CE . Adolescent idiopathic scoliosis screening: could a school-based assessment protocol be useful for an early diagnosis? J Back Musculoskelet Rehabil. (2021) 34:301–6. doi: 10.3233/bmr-200215, 33285626

[7] Zou Y Lin Y Meng J Li J Gu F Zhang R. The prevalence of scoliosis screening positive and its influencing factors: a school-based cross-sectional study in Zhejiang Province, China. Front Public Health. (2022) 10:773594. doi: 10.3389/fpubh.2022.773594, 35923961 PMC9339673

[8] Yaszay B Bastrom TP Bartley CE Parent S Newton PO. The effects of the three-dimensional deformity of adolescent idiopathic scoliosis on pulmonary function. Eur Spine J. (2017) 26:1658–64. doi: 10.1007/s00586-016-4694-y, 27514676

[9] Horne JP Flannery R Usman S. Adolescent idiopathic scoliosis: diagnosis and management. Am Fam Physician. (2014) 89:193–8. 24506121

[10] Bondar K Nguyen A Vatani J Kessler J. The demographics and epidemiology of infantile, juvenile, and adolescent idiopathic scoliosis in a Southern California integrated health care system. Spine (Phila Pa 1976). (2021) 46:1468–77. doi: 10.1097/brs.0000000000004046, 33813584

[11] Monticone M Ambrosini E Cazzaniga D Rocca B Ferrante S. Active self-correction and task-oriented exercises reduce spinal deformity and improve quality of life in subjects with mild adolescent idiopathic scoliosis. Results of a randomised controlled trial. Eur Spine J. (2014) 23:1204–14. doi: 10.1007/s00586-014-3241-y, 24682356

[12] Fu X Meng S Huang X Li W Ye B Chen S. The prevalence of scoliosis among adolescents in China: a systematic review and meta-analysis. J Orthop Surg Res. (2024) 19:585. doi: 10.1186/s13018-024-05077-0, 39342221 PMC11437733

[13] Xu S Su Y Wang Z Liu C Jin L Liu H. Prevalence characteristics of scoliosis among primary and secondary school students in mainland China: a meta-analysis of 72 studies. China Spinal Cord J. (2021) 31:901–10. doi: 10.3969/j.issn.1004-406X.2021.10.05

[14] Zhou L Yang H Hai Y Hai JJ Cheng Y Yin P . Scoliosis among children in Qinghai-Tibetan plateau of China: a cross-sectional epidemiological study. Front Public Health. (2022) 10:983095. doi: 10.3389/fpubh.2022.983095, 36062094 PMC9437421

[15] Guo H Chen N Yang Y Zhou X Li X Jiang Y . Ethnic disparity in the incidence of scoliosis among adolescents in Tianzhu Tibetan Autonomous County, China. Front Public Health. (2022) 10:791550. doi: 10.3389/fpubh.2022.791550, 35570980 PMC9092046

[16] Li T Ma L Yan Y Liu R Sun X Yang Y . Dental caries and associated factors in Tibetan school-age children in Gannan, China. Int Dent J. (2025) 75:643–51. doi: 10.1016/j.identj.2024.09.036, 39675953 PMC11976584

[17] Zhou C Li M Liu L Zhao F Cong W Zhang F. Food consumption and dietary patterns of local adults living on the Tibetan plateau: results from 14 countries along the Yarlung Tsangpo River. Nutrients. (2021) 13:2444. doi: 10.3390/nu13072444, 34371952 PMC8308694

[18] Liu J Xin Z Huang Y Yu J. Climate suitability assessment on the Qinghai-Tibet plateau. Sci Total Environ. (2022) 816:151653. doi: 10.1016/j.scitotenv.2021.151653, 34793809

[19] Pearsall DJ Reid JG Hedden DM. Comparison of three noninvasive methods for measuring scoliosis. Phys Ther. (1992) 72:648–57. doi: 10.1093/ptj/72.9.648, 1508972

[20] de Assis SJC Sanchis GJB de Souza CG Roncalli AG. Influence of physical activity and postural habits in schoolchildren with scoliosis. Arch Public Health. (2021) 79:63. doi: 10.1186/s13690-021-00584-6, 33926556 PMC8086061

[21] Margalit A McKean G Constantine A Thompson CB Lee RJ Sponseller PD. Body mass hides the curve: thoracic scoliometer readings vary by body mass index value. J Pediatr Orthop. (2017) 37:e255–60. doi: 10.1097/bpo.0000000000000899, 27861214 PMC5422115

[22] Qian G Zhang L Zhao Z Wang Y Lu J Bi N . Prevalence of scoliosis and congenital heart disease based on school screening in Jinghong City, Yunnan Province. Front Public Health. (2025) 13:1517542. doi: 10.3389/fpubh.2025.1517542, 40071112 PMC11893419

[23] Ueno M Takaso M Nakazawa T Imura T Saito W Shintani R . A 5-year epidemiological study on the prevalence rate of idiopathic scoliosis in Tokyo: school screening of more than 250,000 children. J Orthop Sci. (2011) 16:1–6. doi: 10.1007/s00776-010-0009-z, 21293892

[24] Singh H Shipra Sharma V Sharma I Sharma A Modeel S . The first study of epidemiology of adolescent idiopathic scoliosis shows lower prevalence in females of Jammu and Kashmir, India. Am J Transl Res. (2022) 14:1100–6. 35273713 PMC8902575

[25] Wong HK Hui JH Rajan U Chia HP. Idiopathic scoliosis in Singapore schoolchildren: a prevalence study 15 years into the screening program. Spine. (2005) 30:1188–96. doi: 10.1097/01.brs.0000162280.95076.bb, 15897834

[26] Kuroki H Nagai T Chosa E Tajima N. School scoliosis screening by moiré topography - overview for 33 years in Miyazaki Japan. J Orthop Sci. (2018) 23:609–13. doi: 10.1016/j.jos.2018.03.005, 29628286

[27] Zhao L Jiang X Zhang W Hao L Wu S Zhang Y . Risk factors, lifestyle and prevention among adolescents with spinal curvature abnormality: a cross-sectional study in twenty-four primary and secondary schools in Hangzhou, Zhejiang Province, China. BMC Public Health. (2025) 25:1741. doi: 10.1186/s12889-025-22883-1, 40361073 PMC12070769

[28] Huang J Zhou X Li X Guo H Yang Y Cheong IOH . Regional disparity in epidemiological characteristics of adolescent scoliosis in China: data from a screening program. Front Public Health. (2022) 10:935040. doi: 10.3389/fpubh.2022.935040, 36561865 PMC9764629

[29] Song S He Y Zhen Q Shui T An W Dai L . Large-scale school scoliosis screening in a multi-ethnic, high-altitude region of southwestern China: an epidemiological study of 69,811 children and adolescents. Front Public Health. (2025) 13:1659046. doi: 10.3389/fpubh.2025.1659046, 41211406 PMC12592162

[30] Peng Y Wang SR Qiu GX Zhang JG Zhuang QY. Research progress on the etiology and pathogenesis of adolescent idiopathic scoliosis. Chin Med J. (2020) 133:483–93. doi: 10.1097/cm9.0000000000000652, 31972723 PMC7046244

[31] Inoue M Minami S Nakata Y Kitahara H Otsuka Y Isobe K . Association between estrogen receptor gene polymorphisms and curve severity of idiopathic scoliosis. Spine (Phila Pa 1976). (2002) 27:2357–62. doi: 10.1097/00007632-200211010-00009 12438984

[32] Qui Y Qiu XS Sun X Wang B Yu Y Zhu ZZ . Body mass index in girls with adolescent idiopathic scoliosis. Zhonghua Wai Ke Za Zhi. (2008) 46:588–91. doi: 10.3321/j.issn:0529-5815.2008.08.009 18844053

[33] Brazolino MAN Maia TC Junior CJ Cardoso IM Junior JLB Batista PR. Low body mass in patients with adolescent idiopathic scoliosis. Salus J Health Sci. (2015) 1:82–6.

[34] Tam EMS Liu Z Lam TP Ting T Cheung G Ng BKW . Lower muscle mass and body fat in adolescent idiopathic scoliosis are associated with abnormal leptin bioavailability. Spine (Phila Pa 1976). (2016) 41:940–6. doi: 10.1097/brs.0000000000001376, 26656046

[35] Jeon K Kim DI. The association between low body weight and scoliosis among Korean elementary school students. Int J Environ Res Public Health. (2018) 15:2613. doi: 10.3390/ijerph15122613, 30469502 PMC6313767

[36] Wei-Jun W Xu S Zhi-Wei W Xu-Sheng Q Zhen L Yong Q. Abnormal anthropometric measurements and growth pattern in male adolescent idiopathic scoliosis. Eur Spine J. (2012) 21:77–83. doi: 10.1007/s00586-011-1960-x, 21826498 PMC3252435

[37] Kim S Uhm JY Chae DH Park Y. Low body mass index for early screening of adolescent idiopathic scoliosis: a comparison based on standardized body mass index classifications. Asian Nurs Res. (2020) 14:24–9. doi: 10.1016/j.anr.2019.12.003, 31923468

[38] Clark EM Taylor HJ Harding I Hutchinson J Nelson I Deanfield JE . Association between components of body composition and scoliosis: a prospective cohort study reporting differences identifiable before the onset of scoliosis. J Bone Miner Res. (2014) 29:1729–36. doi: 10.1002/jbmr.2207, 24616164

[39] Worthington V Shambaugh P. Nutrition as an environmental factor in the etiology of idiopathic scoliosis. J Manip Physiol Ther. (1993) 16:169–73. 8492060

[40] Normand E Franco A Marcil V. Nutrition and physical activity level of adolescents with idiopathic scoliosis: a narrative review. Spine J. (2020) 20:785–99. doi: 10.1016/j.spinee.2019.11.012, 31783126

[41] Yu HG Zhang HQ Zhou ZH Wang YJ. High ghrelin level predicts the curve progression of adolescent idiopathic scoliosis girls. Biomed Res Int. (2018) 2018:9784083. doi: 10.1155/2018/9784083, 30079352 PMC6069699

[42] Liang ZT Guo CF Li J Zhang HQ. The role of endocrine hormones in the pathogenesis of adolescent idiopathic scoliosis. FASEB J. (2021) 35:e21839. doi: 10.1096/fj.202100759R, 34387890

[43] Reid IR Baldock PA Cornish J. Effects of leptin on the skeleton. Endocr Rev. (2018) 39:938–59. doi: 10.1210/er.2017-00226, 30184053

[44] Man GC Tam EM Wong YS Hung VW Hu Z Lam TP . Abnormal osteoblastic response to leptin in patients with adolescent idiopathic scoliosis. Sci Rep. (2019) 9:17128. doi: 10.1038/s41598-019-53757-3, 31748652 PMC6868007

[45] Wang Q Wang C Hu W Hu F Liu W Zhang X. Disordered leptin and ghrelin bioactivity in adolescent idiopathic scoliosis (AIS): a systematic review and meta-analysis. J Orthop Surg Res. (2020) 15:502. doi: 10.1186/s13018-020-01988-w, 33121521 PMC7596938

[46] Krum SA. Direct transcriptional targets of sex steroid hormones in bone. J Cell Biochem. (2011) 112:401–8. doi: 10.1002/jcb.22970, 21268060 PMC3070194

[47] Wu J Qiu Y Zhang L Sun Q Qiu X He Y. Association of estrogen receptor gene polymorphisms with susceptibility to adolescent idiopathic scoliosis. Spine (Phila Pa 1976). (2006) 31:1131–6. doi: 10.1097/01.brs.0000216603.91330.6f, 16648749

[48] Grivas TB Vasiliadis E Savvidou O Mouzakis V Koufopoulos G. Geographic latitude and prevalence of adolescent idiopathic scoliosis. Stud Health Technol Inform. (2006) 123:84–9. 17108408

[49] Ladizesky MG Boggio V Albornoz LE Castrillón PO Mautalen C Cardinali DP. Melatonin increases oestradiol-induced bone formation in ovariectomized rats. J Pineal Res. (2003) 34:143–51. doi: 10.1034/j.1600-079x.2003.00021.x, 12562506

[50] Zhang K Xu N Guo C Wu J. MPF-net: an effective framework for automated cobb angle estimation. Med Image Anal. (2022) 75:102277. doi: 10.1016/j.media.2021.102277, 34753020

[51] Larson JE Meyer MA Boody B Sarwark JF. Evaluation of angle trunk rotation measurements to improve quality and safety in the management of adolescent idiopathic scoliosis. J Orthop. (2018) 15:563–5. doi: 10.1016/j.jor.2018.05.032, 29881194 PMC5990317

[52] Chen C Yu R Xu W Li Z Li Y Hu R . A practical study of diagnostic accuracy: scoliosis screenings of middle school students by a trained nurse with a smartphone versus a spine surgeon with a scoliometer. Spine (Phila Pa 1976). (2020) 45:E266–e271. doi: 10.1097/brs.0000000000003256, 31568349

[53] Ronckers CM Doody MM Lonstein JE Stovall M Land CE. Multiple diagnostic X-rays for spine deformities and risk of breast cancer. Cancer Epidemiol Biomarkers Prev. (2008) 17:605–13. doi: 10.1158/1055-9965.Epi-07-2628, 18349278

[54] Du X Bai T Lv C Zhang Z Dong X Yang W . Epidemiological trends and predictive factors of adolescent scoliosis in Qingdao: insights from a large-scale screening program (2022-2024). Eur Spine J. (2025) 34:5271–9. doi: 10.1007/s00586-025-09245-6, 40804499

